# Two New Phenolic Constituents from the Stems of *Euphorbia griffithii*

**DOI:** 10.1007/s13659-019-00223-2

**Published:** 2019-11-16

**Authors:** Joseph Sakah Kaunda, Ying-Jun Zhang

**Affiliations:** 1 State Key Laboratory of Phytochemistry and Plant Resources in West China, Kunming Institute of Botany, Chinese Academy of Sciences, Kunming, 650204 People’s Republic of China; 2 Graduate School of the Chinese Academy of Sciences, Beijing, 100039 People’s Republic of China; 3 Yunnan Key Laboratory of Natural Medicinal Chemistry, Kunming Institute of Botany, Chinese Academy of Sciences, Kunming, 650201 People’s Republic of China

**Keywords:** Euphorbiaceae, *Euphorbia griffithii*, Phenolic glycosides, Hydrolyzable tannins

## Abstract

**Abstract:**

Phytochemical studies on MeOH extract of stems of *Euphorbia griffithii* led to the isolation of one new hydrolyzable tannin dimer, corilagiffithiin (**1**) and one new galloyl-glucoside (**2**), alongside six known ones (**3**–**8**). Their structures and absolute configurations were determined by in depth spectroscopic analyses and comparison of their 1D NMR and MS data with literature reported values. Configurations of sugar moieties were determined by acidic hydrolysis and subsequent GC analysis of their corresponding trimethylsilylated l-cysteine adduct. At a concentration of 50 μM, compounds **1**–**3** showed no anti-inflammatory activities.

**Graphic Abstract:**

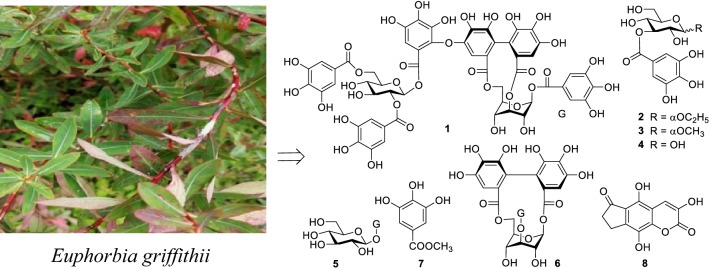

**Electronic supplementary material:**

The online version of this article (10.1007/s13659-019-00223-2) contains supplementary material, which is available to authorized users.

## Introduction

Among the flowering plants, the genus *Euphorbia* (spurges, Euphorbiaceae) is the third largest with almost 2000 species spread throughout the world [1–3]. They inhabit a wide range of habitats and display immense diversity in growth patterns. In their environment, the species vary from small ephemerals to diversified forms of herbaceous annuals, cushion-forming subshrubs, large shrubs, small trees and cactus-like succulents [2]. Spurges often produce toxic white latex when cut [1]. Different communities utilize these plants to treat various kinds of diseases. In India, many species are applied in the management of asthma and respiratory tract inflammations. In Angola, some species are used against skin ailments, gonorrhea, diarrhea, dysentery, asthma, tumors and coughs. In Nigeria, the exudates of the plant are employed as ear drops, in the treatment of boils, sores and enhancement of wound healing [1]. Spurges are also used as ornamental and household plants [1]. Phytochemical investigations have revealed that the *Euphorbia* genus contains mainly, triterpenoids, diterpenoids, flavonoids, tannins and polyphenols [1, 3]. Pharmacologically, the diterpenoids of *Euphorbia* have exhibited cytotoxic activities, while the tripernoids and flavonoids have demonstrated possession of anti-inflammation and inhibition of virus replication effects [1, 3, 4].

*E. griffithii* is a robust rhizomatous perennial whose vertical stems dresses in small, red-tinged leaves and showy orange-red flowers in early summer; it is native to Himalayas and western Asia [5, 6]. It has red stems and dark green leaves, which crop up in spring with a reddish tincture [7]. Exhaustive literature search did not show any evidence of previous phytochemical studies on *E. griffithii*. That notwithstanding, our study on the stems of *E. griffithii* disclosed the presence of one new hydrolyzable tannin dimer (**1**) and one new galloyl-glucoside (**2**), together with six known ones (**3**–**8**). Compounds **1**–**3** were evaluated for their anti-inflammatory effects. Herein, we present the isolation, structural elucidation of the isolates, and bioassay tests of compounds **1**–**3**.

## Results, Discussion and Conclusion

**Fig. 1:**
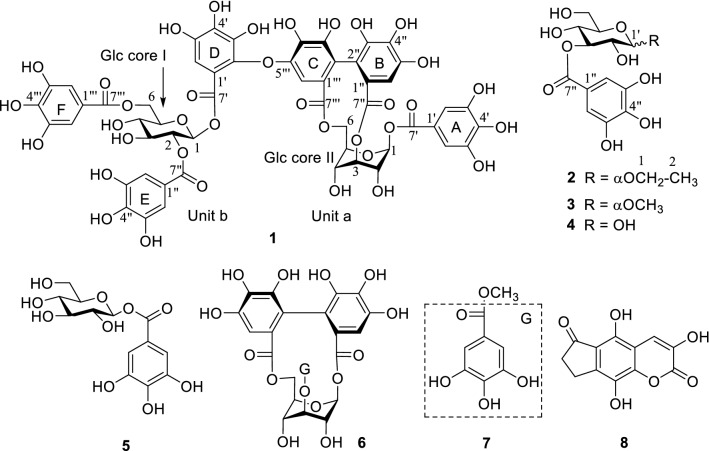
Chemical structures of **1**–**8** from *E. griffithii*

Phytochemical investigation of the stems of *E. griffithii*, by using various column chromatography, afforded two new compounds (**1**–**2**) (Fig. 1). In addition, six known compounds (**3**–**8**) were isolated and determined, on the basis of comparison of their NMR spectra and MS data with those reported in the literature, as methyl-​*O*-(3′*-O*-galloyl)-*α*-d (**3**) [8], 3-*O*-galloyl-d-glucose (**4**) [9], *β*-d-glucogallin (**5**) [10], helioscopinin B (**6**) [11], methyl gallate (**7**) [12], and rubusin A (**8**) [13] (Fig. 1), respectively.

**Fig. 2:**
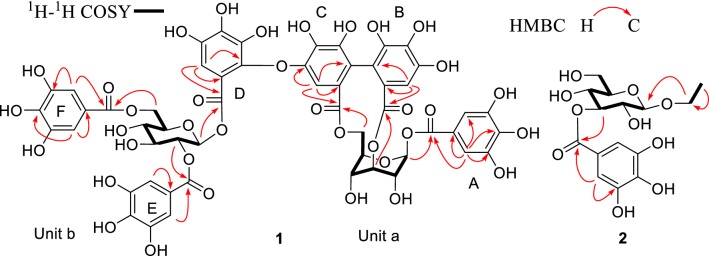
Key^1^-^1^H COSY and HMBC correlations of **1**–**2**

Corilagiffithiin (**1**) was isolated as a dark brown solid. In HRFAB^+^, a quasi-molecular ion peak appeared at *m/z* 1291.1508 [M + Na]^+^ (calcd for 1291.1510 [M + Na]^+^), indicating a molecular formula of C_54_H_44_O_36_ for **1**. The ^1^H NMR spectrum displayed signals corresponding to three galloyl moieties (*δ*_H_ 7.14, 7.10, 7.09, each 2H, s), and one hexahydroxydiphenoyl (HHDP) group (*δ*_H_ 6.91, 6.25, each 1H, s). Moreover, ^1^H NMR spectrum showed a proton signal at *δ*_H_ 7.10 (1H, s), suggestive of the existence of an additional aromatic group. Another characteristic feature of the ^1^H NMR spectrum was the presence of two glucosyl proton signals at *δ*_H_ 5.81 (1H, d, *J* = 9.0 Hz,), as well as at *δ*_H_ 6.36 (1H, d, *J* = 2.0 Hz), which, on the basis of acidic hydrolysis and subsequent GC analysis of their corresponding trimethylsilylated l-cysteine adduct, were determined to be d-glucosyl units. The ^13^C NMR spectrum showed a total of 54 signals, some of which, on grounds of HMBC experiment, confirmed the presence of three galloyl [*δ*_C_ 120.9, 110.8 × 2, 146.0 × 2, 140.0, 165.26 (ring A, C-1′-7′, resp.); *δ*_C_ 120.2, 110.8 × 2, 146.1 × 2, 139.4, 165.33 (ring E, C-1″-7″, resp.); *δ*_C_ 121.6, 110.4 × 2, 146.2 × 2, 139.0, 166.8 (ring F, C-1″′-7″′, resp.)], one HHDP [*δ*_C_ 125.2, 117.0, 144.8, 137.4, 144.9, 110.0, 167.4 (Unit a, C-1″-7″, resp.); *δ*_C_ 125.3, 117.7, 145.6, 136.9, 147.5, 104.8, 168.5 (Unit a, C-1″′-7″′, resp.)]; and one aromatic [*δ*_C_ 115.3, 143.6, 140.6, 135.8, 139.8, 110.8, 165.7 (Unit b, C-1′-7′, resp.)] groups. Following the guidance of ^1^H-^1^H COSY experiment, six carbon signals (*δ*_C_ 94.5, 69.4, 70.9, 62.4, 75.6 and 64.4) were assigned to the ^1^C_4_ glucopyranose core II, while six other signals (*δ*_C_ 93.9, 73.7, 75.7, 71.3, 75.2 and 63.9) were allocated to the ^4^C_1_ glucopyranose core I [14]. In the HMBC experiment of **1**, correlations of *δ*_H_ 6.36 (H-1) and *δ*_H_ 7.10 (H-2′,6′) with *δ*_C_ 165.26 (C-7′) in Unit a; and *δ*_H_ 5.18 (H-2) and *δ*_H_ 7.09 (H-2″,6″) with *δ*_C_ 165.33 (C-7″), and *δ*_H_ 4.53 (H-6) and *δ*_H_ 7.14 (C-2″′,6″′) with *δ*_C_ 166.8 (C-7″′) in Unit b were observed, indicating that the hydroxy groups at C-1 of Unit a and C-2, C-6 of Unit b were acylated by galloyl (A, E and F) groups, resp. (Fig. 2). Location of the HHDP group at O-3/O-6 of the glucose moiety of unit a was revealed by the down-field shifted proton signals of glucosyl H-3 (*δ*_H_ 4.89) and H_2_-6 [*δ*_*H*_ 4.71, 4.15] and established by HMBC correlations [H-3 (*δ*_H_ 4.89) and H-6″ (*δ*_H_ 6.91) correlated with *δ*_C_ 167.4 (C-7″); H-6a (*δ*_H_ 4.71), H-6b (*δ*_H_ 4.15) and H-6″′ (*δ*_H_ 6.25) correlated with C-7″′ (*δ*_C_ 168.5)]. On Unit b, HMBC showed correlations from *δ*_H_ 5.81 (H-1) and *δ*_H_ 7.10 (H-6′) with *δ*_C_ 165.7 (C-7′), and from H-6′ (*δ*_H_ 7.10) to C-1′ (*δ*_C_ 115.3), C-2′ (*δ*_C_ 143.6), C-4′ (*δ*_C_ 135.8), and C-5′ (*δ*_C_ 139.8) of the aromatic moiety (ring D) (Fig. 2). Eventually, the planar structures of Units a and b were determined (Fig. 1). Unit a was found matched perfectly corilagin [15] while the structure of unit b was quite similar to 1,2,6-tri-*O*-galloyl-*β*-d-glucopyranose [16], by comparisons of their spectral data.

The linkage position between the two units, a and b, was determined by the ^13^C NMR chemical shift values and HMBC correlations. Firstly, HMBC correlations of H-6 and H-6″′ with C-7″′ in unit a, and H-1 and H-6′ with C-7′ in unit b confirmed the locations of aromatic rings C and D on O-6 of Glc-II and O-1 of Glc-I, respectively. The presence of five quaternary carbons [*δ*_C_ 115.3, 143.6, 140.6, 135.8, and 139.8 (C-1′-5′, resp.) in aromatic ring D suggested that it was a galloyl group that had undergone oxidative coupling at C-2′, which was significantly downfield shifted to *δ*_C_ 143.6, hence taking part in an ether linkage. Moreover, it was observed that the ^13^C NMR resonance of C-5″′ of the HHDP moiety (ring C) was shifted further downfield (*δ*_C_ 147.5), lower than the usual corresponding C-5″′ signal (*δ*_C_ 145.6) of the corilagin [17, 18]. This observation was attributed to formation of an ether linkage at the *p*-hydroxyl group of the galloyl unit [17]. The HMBC spectrum also showed correlations of H-6″′ (*δ*_H_ 6.25) with C-5″′ (*δ*_C_ 147.5) of the HHDP ring C. Eventually, the structure of compound **1**, namely corilagiffithiin was determined as shown in Fig. 1.

Ethyl-*O*-(3′*-O*-galloyl)-*α*-d-glucopyranoside (**2**) was obtained as a dark brown solid. Its molecular formula, C_15_H_20_O_10_, was determined based on the HRESIMS quasi molecular ion peak at *m/z* 359.0987 (calcd for C_15_H_20_O_10_ [M−H]^−^, 359.0984). The ^1^H NMR spectrum displayed signals attributable to one methyl at *δ* 1.25 (1H, t, *J* = 7.3 Hz, H-2), one *α*-anomeric proton signal at *δ* 4.87 (1H, d, *J* = 3.8 Hz, H-1′), and one set of two methines belonging to a galloyl moiety at *δ* 7.12 (2H, s, H-2″,6″). The ^13^C NMR spectrum showed 14 signals signifying the existence of a methyl (*δ*_C_ 15.3), a glucopyranosyl moiety at 99.6, 77.2, 73.3, 73.3, 71.8, 62.3, and a galloyl moiety [ester carbonyl carbon (*δ*_C_ 168.5), a set of two oxygenated quaternary carbons (*δ*_C_ 146.2), and two quaternary carbons (*δ*_C_ 139.6 and 121.7). Moreover, in the ^1^H–^1^H COSY spectrum, the proton sequence pattern revealed the presence of a glucopyranose moiety which, owing to the small coupling constant of the anomeric proton [*δ* 4.87 (1H, d, *J* = 3.8 Hz, H-1′)] and acidic hydrolysis, was suggested to be *α-*d-glucopyranosyl moiety [19, 20]. ^1^H–^1^H COSY [*δ*_H_ 1.25 (H-2) / *δ*_H_ 3.76 (H-1a), 3.48 (H-1b)], jointly with HMBC correlation [from *δ*_H_ 1.25 (H-2) to *δ*_C_ 64.6 (C-1)] supported the existence of an ethoxyl group in **2**. HMBC experiment showed that the protons at *δ* 5.33 (H-3′ of the glucose moiety) and 7.12 (H-2″ and 6″ of the galloyl group) correlated with *δ*_C_ 168.5 (the carbonyl carbon of the galloyl group), connoting that the hydroxyl group at C-3′ of glucose was acylated by the galloyl group (Fig. 3). A closer look at the planar structure of compound **2** showed that it was constituted of a galloyl [21] and ethyl *α*-D-glucopyranoside [22]. Thus, compound **2** was identified as ethyl-*O*-(3′*-O*-galloyl)-*α*-d-glucopyranoside.

Compounds **1**–**3** were evaluated for their anti-inflammatory effects on NO levels in LPS-stimulated RAW 264.7 macrophages. The compounds were inactive under the concentration of 50 μM.

In conclusion, from the methanolic stem extract of *E. griffithii*, compounds **1**–**8**, comprising two new ones (**1**–**2**) and six known ones (**3**–**8**), were isolated. Compound **1** is a hydrolysable tannin possessing corilagin linked with 1,2,6-tri-*O*-galloyl-*β*-d-glucopyranose, whereas **2** constituted of a galloyl and ethyl-d-glucopyranoside. However, it is important to emphasize that compound **3** was isolated, for the first time, as a natural product. The only mention of compound **3** was by Masayuki et al. [20] and Arapitsas P. et al. [8]. Masayuki et al. [20] obtained compound **3** as a byproduct from acid hydrolysis of castanopsinin G (an ellagitannin containing triterpenoid glycoside core) with 1 N methanolic sulphuric acid [20]. None of the tested compounds **1**–**3**, exhibited anti-inflammatory activities.

## Experimental Section

### General Experimental Procedure

UV spectra were recorded on a UV 210A Shimadzu spectrometer. CD spectra were obtained on a JASCO 810 spectrometer. IR spectra were measured on a Bio-Rad FTS- 135 series spectrometer with KBr pellets. Optical rotations were measured on Rudolph Autopol VI polarimeter (Rudolph Research Analytical, Hacketstown, NJ, USA). One- and two-dimensional (1D and 2D) NMR spectra were recorded in acetone-*d*_6_ with Avance 600 spectrometer operating at 600 MHz for ^1^H and at 150 MHz for ^13^C. Coupling constants are expressed in hertz, and chemical shifts are given on a *δ* (parts per million, ppm) scale with tetramethylsilane (TMS) as an internal standard. ESI mass spectra were recorded on a VG Auto Spec-300 spectrometer. High-resolution (HR) ESI mass spectra were recorded on an API QSTAR Pular-1 spectrometer. FAB and HRFAB were measured on Agilent G6230 TOF MS. Anti-inflammatory activity was evaluated on the basis of the ability of the compounds to inhibit nitric oxide (NO) production in LPS-stimulated RAW 264.7 macrophages. Column chromatography was performed on Sephadex LH-20 (25 − 100 μm Pharmacia Fine Chemical Co., Ltd.), MCI gel CHP20P (75 − 100 μm, Mitsubishi Chemical Co., Ltd.) and Toyopearl HW-40F (Tosoh Co., Ltd.). Thin-layer chromatography (TLC) was performed on precoated silica gel plates, 0.20 − 0.25 mm thick (Qingdao Haiyang Chemical Co.), with benzene/ethyl formate/formic acid (1:7:1, 2:7:1, or 3:6:1 v/v/v) or chloroform/methanol/water (7:3:0.5 or 8:2:0.2 v/v/v), and spots were visualized by spraying with 2% ethanolic FeCl_3_ or 10% H_2_SO_4_ in EtOH, followed by heating. Water was purified in a Milli-Q (Millipore, America). Acetonitrile (chromatographic grade) was purchased from Merck (Darmstadt, FR, Germany).

### Plant Material

Stems of *E. griffithii* were collected in in July 2017 from the vicinity of Baisha township, Yulong county, Lijiang, Yunnan province, China, and identified by Prof. C.R. Yang from Kunming Institute of Botany (KIB), Chinese Academy of Sciences (CAS). A voucher specimen (KIB-Z-2017008) was deposited in the State Key Laboratory of Phytochemistry and Plant Resource in West China of KIB, CAS.

### Extraction and Isolation

The air-dried stems of *E. griffithii* (10 kg) were extracted with MeOH (three times) under reflux at 60 °C. After removal of the organic solvent, the extract (4.5 kg) was partitioned with H_2_O and EtOAc to generate H_2_O extract (1.5 kg) and EtOAc extract (1.2 kg). EtOAc extract was applied to Sephadex LH-20 column, eluting with EtOH to give 11 sub fractions 2.1–2.11. Fr. 2.2 (15.8 g), fr. 2.6 (9.2 g), fr. 2.7 (15.0 g) and fr. 2.8 were separately chromatographed over MCI gel CHP20P [MeOH: H_2_O (0:1 → 1:0)] to give compounds **3** (39 mg) and **4** (33 mg) from fr. 2.2, **5** (70 mg) from fr. 2.6, and **6** (12 mg) from fr. 2.7, respectively. The water layer was submitted to Sephadex LH-20 column, eluting with MeOH: H_2_O (0:1 → 1:0), to afford 10 sub fractions, fr. 3.1–3.10. Fr. 3.3 (1.2 g) was applied to Toyopearl HW-40F, eluting with MeOH: H_2_O (0:1 → 1:0) to yield compounds **7** (502 mg) and **8** (62 mg). Similarly, fr. 3.8 (1.2 g) was submitted to Toyopearl HW-40F, eluting with MeOH: H_2_O (0:1 → 1:0; acetone) to yield compounds **1** (534 mg) and **2** (360 mg).

#### Compound 1

Dark brown solid (MeOH); [*α*] −79.7 (*c* 0.1, MeOH); UV (MeOH) *λ*_max_ (log *ε*) 276 (2.34), 248 (1.54), 219 (4.87) nm; IR (KBr) *ν*_max_ 3856, 3405, 1717, 1616, 1532, 1512, 1449, 1350, 1212, 1074, 1036, 971, 872, 837, 765, 663, 620; ^1^H NMR (acetone-*d*_5_, 600 MHz) *δ*_H_ Unit a: 7.10 (2H, s, H-2′,6′), 6.91 (1H, s, H-6″′), 6.36 (1H, d, *J* = 2.0 Hz, H-1), 6.25 (1H, s, H-6″′), 4.89 (1H, br s, H-3), 4.71 (1H, t, *J* = 11.0 Hz, H-6a), 4.47 (overlapped H-4, 5), 4.15 (1H, dd, *J* = 11.0, 8.2 Hz, H-6b), 4.10 (1H, br s, H-2), Unit b: 7.14 (2H, s, H-2″′,6″′), 7.10 (1H, s, H-6′), 7.09 (2H, s, H-2″,6″), 5.81 (1H, d, *J* = 9.0 Hz, H-1), 5.18 (1H, dd, *J* = 9.6, 8.4 Hz, H-2), 4.53 (1H, dd, *J* = 12.2, 1.9 Hz, H-6a), 4.47 (1H, dd, *J* = 12.2, 1.9 Hz, H-6b), 3.89 (1H, m, H-5), 3.67 (1H, t, *J* = 9.1 Hz, H-4), 3.58 (1H, t, *J* = 9.4 Hz, H-3); ^13^C NMR (acetone-*d*_5_, 150 MHz) *δ*_*C*_ Unit a: 168.5 (COO, C-7″′), 167.4 (COO, C-7″), 165.26 (COO, C-7′), 147.5 (C, C-5″′), 146.0 × 2 (C, C-3′, 5′), 145.6 (C, C-3″′), 144.9 (C, C-5″), 144.8 (C, C-3″), 140.0 (C, C-4′), 137.4 (C, C-4″), 136.9 (C, C-4″′), 125.3 (C, C-1″′), 125.2 (C, C-1″), 117.7 (C, C-2″′), 117.0 (C, C-2″), 110.8 × 2 (CH, C-2′,6′), 110.0 (CH, C-6″), 104.8 (CH, C-6″′), 94.5 (CH, C-1), 75.6 (CH, C-5), 70.9 (CH, C-3), 69.4 (CH, C-2), 64.4 (CH_2_, C-6), 62.4 (CH, C-4), Unit b: 166.8 (COO, C-7″′), 165.7 (COO, C-7′), 165.33 (COO, C-7″), 146.2 × 2 (C, C-3″′,5″′), 146.1 × 2 (C , C-3″,5″), 143.6 (C, C-2′), 140.6 (C, C-3′), 139.8 (C, C-5′), 139.4 (C, C-4″′), 139.0 (C, C-4″′), 135.8 (C, C-4′), 121.6 (C, C-1″′), 120.2 (C, C-1″), 115.3 (C, C-1′), 110.8 × 3 (CH, C-6′, 2″,6″), 110.4 × 2 (CH, C-2″′,6″′), 93.9 (CH, C-1), 75.7 (CH, C-5), 75.2 (CH, C-3), 73.7 (CH, C-2), 71.3 (CH, C-4), 63.9 (CH_2_, C-6); HRFAB^+^*m/z* 1291.1508 [M + Na]^+^ (calcd for C_54_H_44_O_36_Na [M + Na]^+^, 1291.1510).

#### Compound 2

Dark brown solid (MeOH); [*α*] 74.0 (*c* 0.1, MeOH); UV (MeOH) *λ*_max_ (log *ε*) 373 (0.01), 371 (0.00), 324 (0.06), 322 (0.06), 275 (1.21), 241 (0.24), 217 (2.96), 200 (1.84) nm; IR (KBr) *ν*_max_ 3385, 2978, 2934, 1701, 1614, 1534, 1448, 1378, 1336, 1232, 1152, 1090, 1039, 960, 887, 815, 767, 751, 700, 645, 602, 545, 472 cm ^−1^; ^1^H NMR (CD_3_OD, 600 MHz) *δ*_*H*_ 7.12 (1H, s, H-2″,6″), 5.33 (1H, t, *J* = 9.6 Hz, H-3′), 4.87 (1H, d, *J* = 3.8 Hz, H-1′), 3.80 (1H, dd, *J* = 11.9, 2.2 Hz, H-6′a), 3.76 (1H, m, H-1a), 3.72 (1H, m, H-6′b), 3.68 (1H, m, H-2′,5′), 3.64 (1H, dd, *J* = 3.7 Hz, 9.9 Hz, H-4′), 3.48 (1H, m, H-1b), 1.25 (1H, t, *J* = 7.3 Hz, H-2); ^13^C NMR (CD_3_OD, 150 MHz) *δ*_*C*_ 168.5 (COO, C-7″), 146.2 (C, C-3″, 5″), 139.5 (C, C-4″), 121.7 (C, C-1″), 110.4 (CH, C-2″, 6″), 99.6 (CH, C-1′), 77.2 (CH, C-3′), 73.3 (CH, C-2′, 5′), 71.8 (CH, C-4′), 64.6 (CH_2_, C-1), 62.3 (CH_2_, CH_2_OH), 15.3 (CH_3_, C-2); ESIMS *m/z* 359 [M + H]^+^; negative ion HRESIMS *m/z* 359.0987 (calcd for C_15_H_20_O_10_ [M−H]^−^, 359.0984).

#### Acid Hydrolysis of Compounds 1–2 and GC Analysis

Compounds, **1**–**2** (3–5 mg), were separately dissolved in 5% HCl (2 mL) and heated (90 °C) for 2 h. HCl was then removed by evaporation in vacuum. The sugar mixtures were diluted with H_2_O and extracted with EtOAc. The aqueous layer was neutralized with 0.1 M NaOH and eventually dried to yield the monosaccharide mixture which was immediately dissolved in pyridine (2 mL). l-cysteine methyl ester hydrochloride (about 1.5 mg) was added in to the solution of the sugar mixtures in pyridine (2 mL), and the reaction kept at 60 °C for 1 h. Thereafter, trimethylsilylimidazole (2 mL) was added in to the mixtures and reaction kept further at 60 °C for 1 h and finally halted then submitted for GC analysis [23], run on Hewlett Packard (HP) 5890 series II gas chromatography equipped with flame ionization detector (FID) and thermal conductivity detector (TCD). The column used was HP-5: column temperature: 150–280 °C, increasing at the rate of: 3 °C/min; carrier gas: N2 (1.5 mL/min); injector and detector temperature: 250 °C; injection volume: 1.0 μL; and split ratio: 1/50. The retention times of the samples were compared with those of the derivatives of authentic sugars, under the same condition. The sugar moiety of **1** was determined to be d-glucose (*t*_R_: 28.537 min), **2** was also resolved to be d-glucose (*t*_R_: 28.398 min), by confirming with the standard d-glucose (*t*_R_: 28.418 min).

#### Anti-Inflammatory Activity

The NO production assay with L-*N*^G^-monomethyl arginine (L-NMMA) as a positive control was performed as described previously by Chen et al. [24].

## Electronic supplementary material

Below is the link to the electronic supplementary material.

Supplementary file1 (DOCX 19899 kb)
