# Supplementary material for: Two New Phenolic Constituents from the Stems of Euphorbia griffithii
Source: Nat Prod Bioprospect. 2019 Nov 16;9(6):405–10. doi: 10.1007/s13659-019-00223-2 (PMC6872693; doi:10.1007/s13659-019-00223-2)
Supplement: Supplementary file 1 — Supplementary file1 (DOCX 19899 kb) [file 13659_2019_223_MOESM1_ESM.docx]

Supporting information

**Two new phenolic constituents from the stems of *Euphorbia griffithii***

Joseph Sakah Kaunda^a,b^ and Ying-Jun Zhang^a,c,*^

*^a^ State Key Laboratory of Phytochemistry and Plant Resources in West China, Kunming Institute of Botany, Chinese Academy of Sciences, Kunming 650204, P. R. China*

*^b^ Graduate School of the Chinese Academy of Sciences, Beijing 100039, P. R. China*

*^c^ Yunnan Key Laboratory of Natural Medicinal Chemistry, Kunming Institute of Botany, Chinese Academy of Sciences, Kunming 650201, People's Republic of China*

**The List of Contents**

| No. | Content |
| --- | --- |
| **S1** | ^1^H NMR spectrum of compound **1** |
| **S2** | ^13^C NMR spectrum of compound **1** |
| **S3** | HSQC spectrum of compound **1** |
| **S4** | HMBC spectrum of compound **1** |
| **S5** | ^1^H-^1^H COSY spectrum of compound **1** |
| **S6** | ESI spectrum of compound **1** |
| **S7** | HRFAB spectrum of compound **1** |
| **S8** | UV spectrum of compound **1** |
| **S9** | IR spectrum of compound **1** |
| **S10** | ^1^H NMR spectrum of compound **2** |
| **S11** | ^13^C NMR spectrum of compound **2** |
| **S12** | HSQC spectrum of compound **2** |
| **S13** | HMBC spectrum of compound**2** |
| **S14** | ^1^H-^1^H COSY spectrum of compound **2** |
| **S15** | ESI spectrum of compound **2** |
| **S16** | HRESI spectrum of compound **2** |
| **S17** | UV spectrum of compound **2** |
| **S18** | IR spectrum of compound **2** |

**Corresponding author:**

Ying-Jun Zhang

*State Key Laboratory of Phytochemistry and Plant Resources in West China, Kunming Institute of Botany, Chinese Academy of Sciences, Kunming 650204, P. R. China*

*Tel/Fax: +86-871-6522-3235. E-mail: [zhangyj@mail.kib.ac.cn](mailto:zhangyj@mail.kib.ac.cn)

**S1** ^1^H NMR spectrum of compound **1**

**S2** ^13^C NMR spectrum of compound **1**

**S3** HSQC spectrum of compound **1**

**S4** HMBC spectrum of compound **1**

**S5** ^1^H-^1^H COSY spectrum of compound **1**


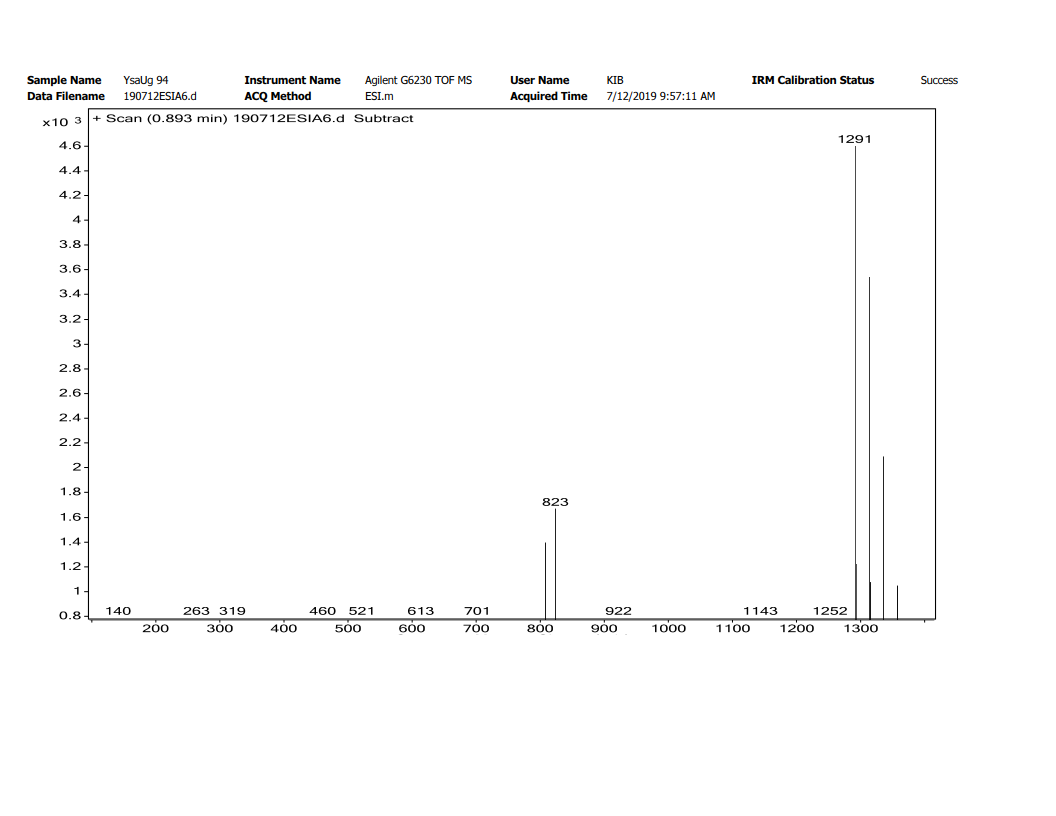


**S6** ESI spectrum of compound **1**


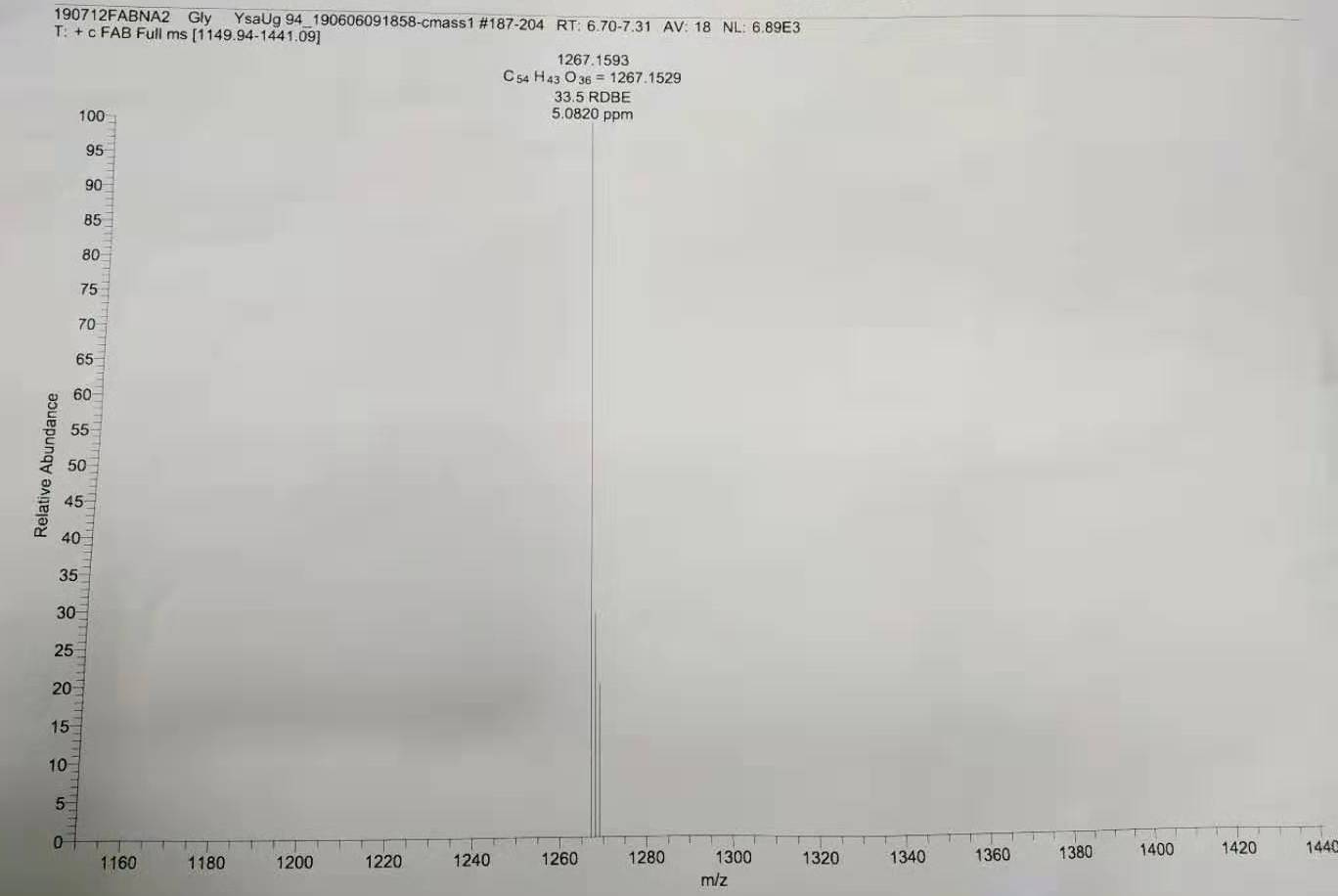


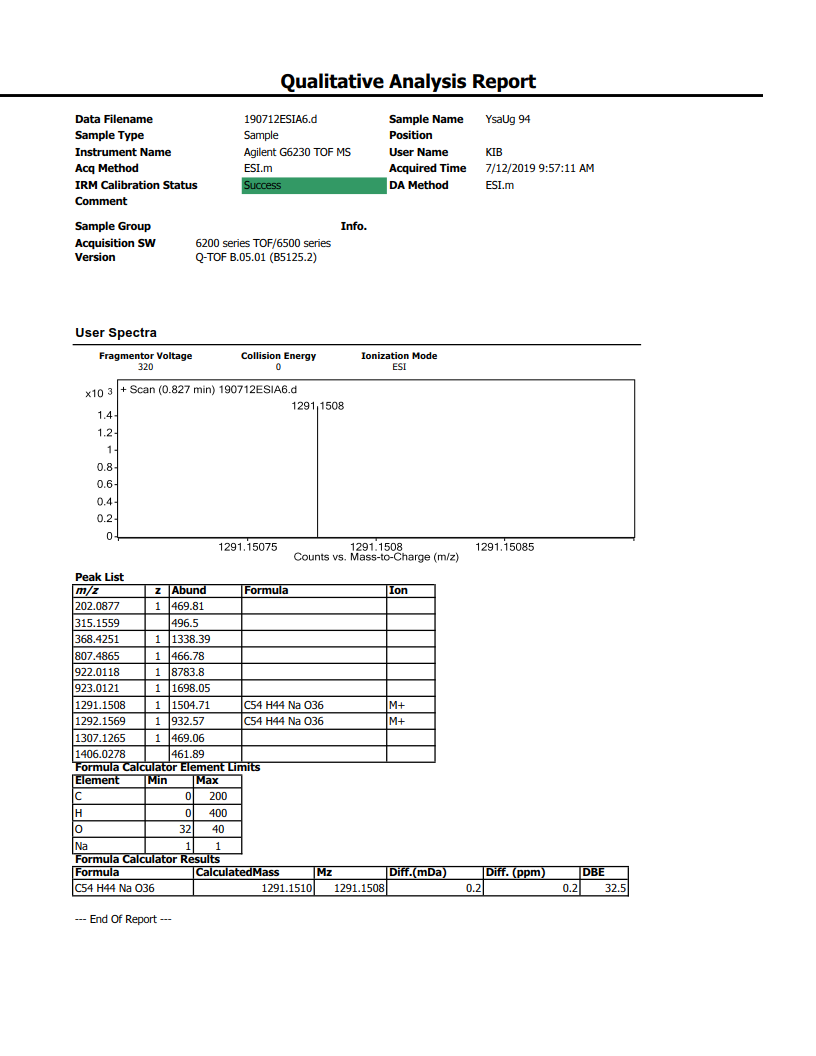


**S7** HRFAB spectrum of compound **1**


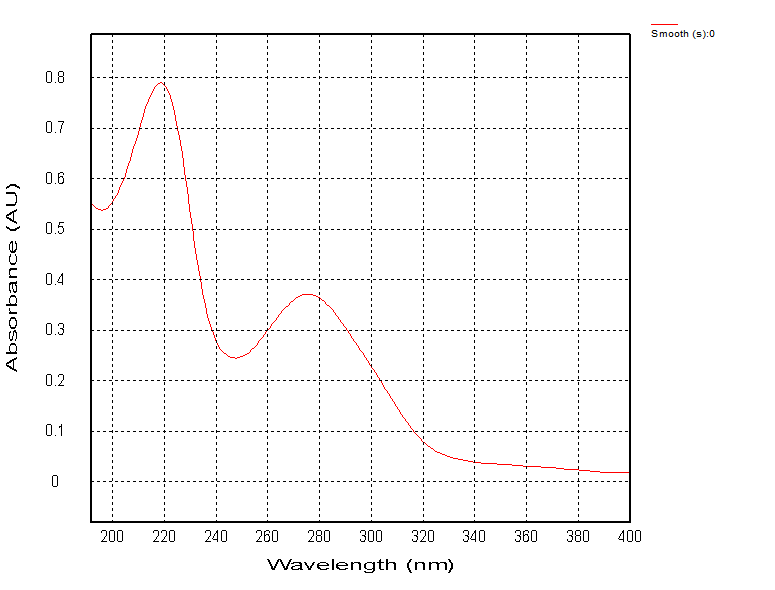


**S8** UV spectrum of compound **1**

**
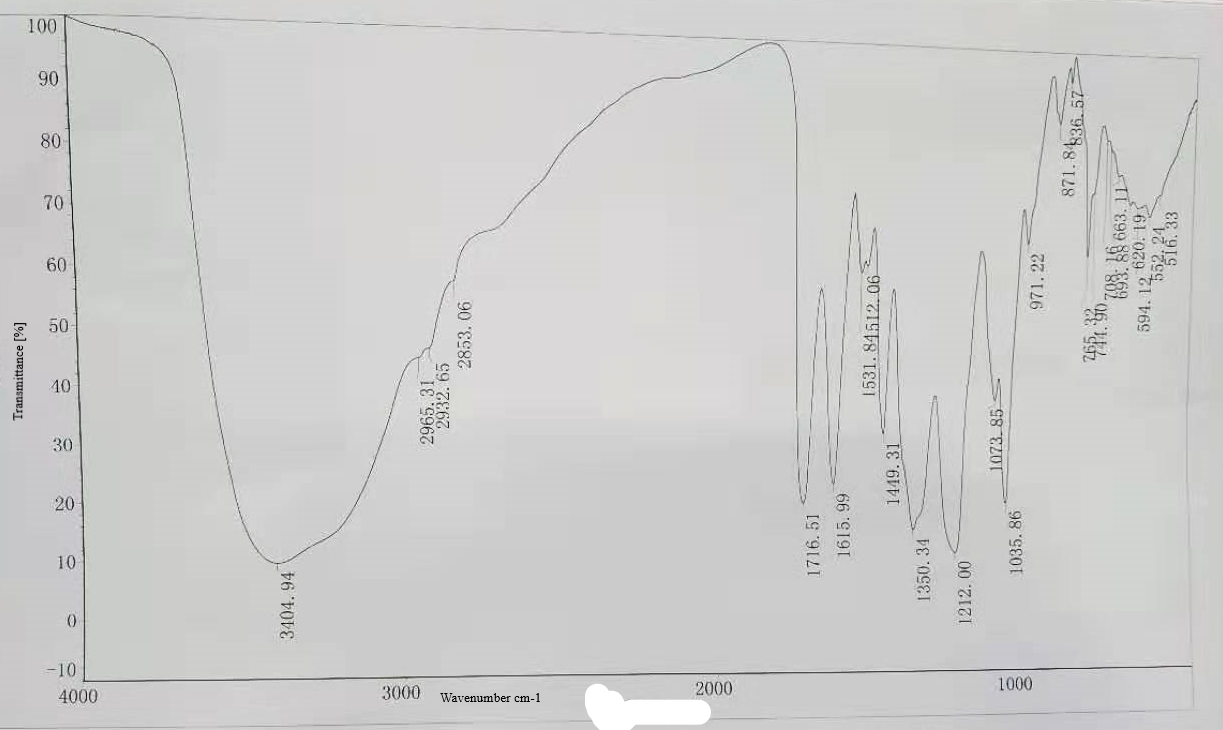
**

**S9** IR spectrum of compound **1**

**S10** ^1^H NMR spectrum of compound **2**

**S11** ^13^C NMR spectrum of compound **2**

**S12** HSQC spectrum of compound **2**

**S13** HMBC spectrum of compound **2**

**S14** ^1^H-^1^H COSY spectrum of compound **2**


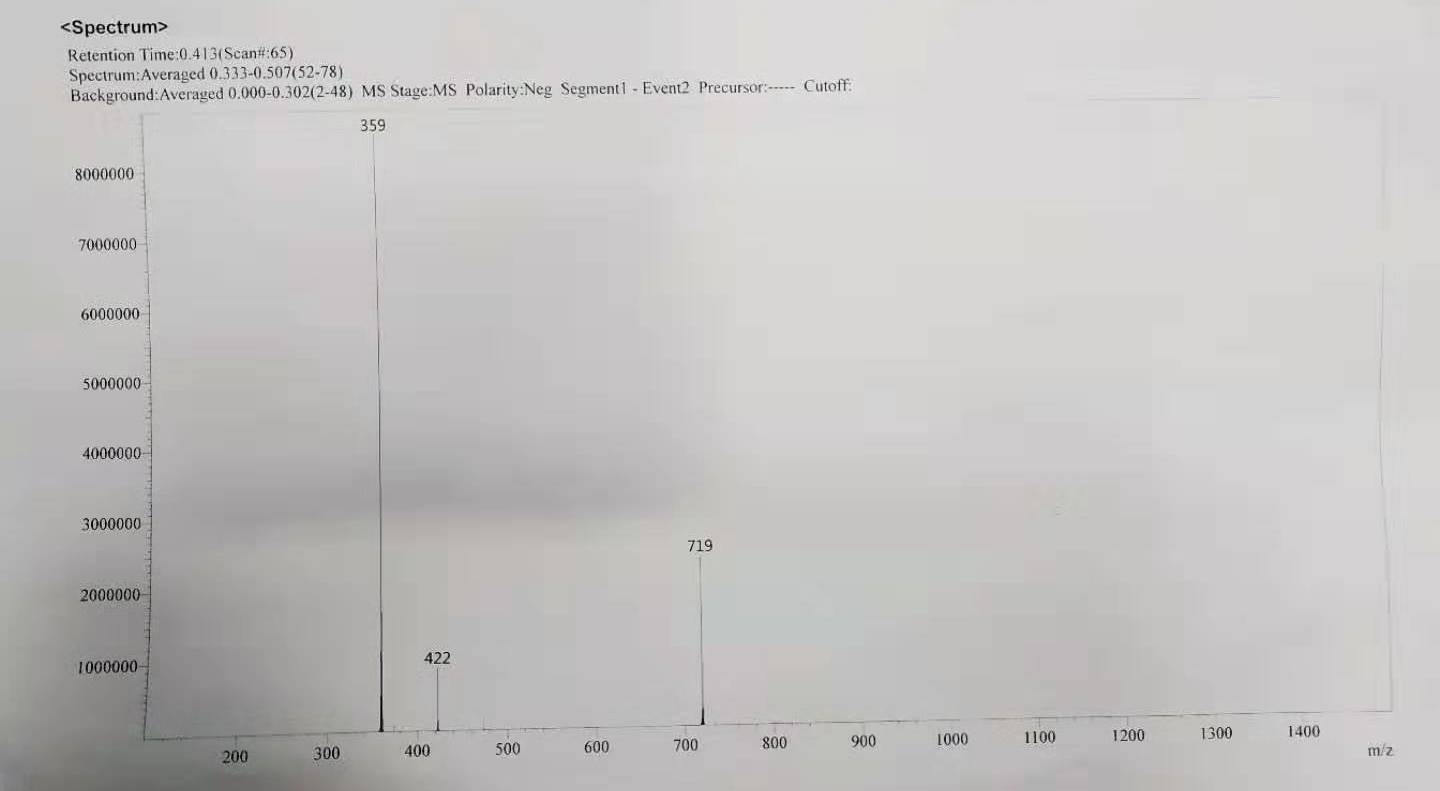


**S15** ESI spectrum of compound **2**


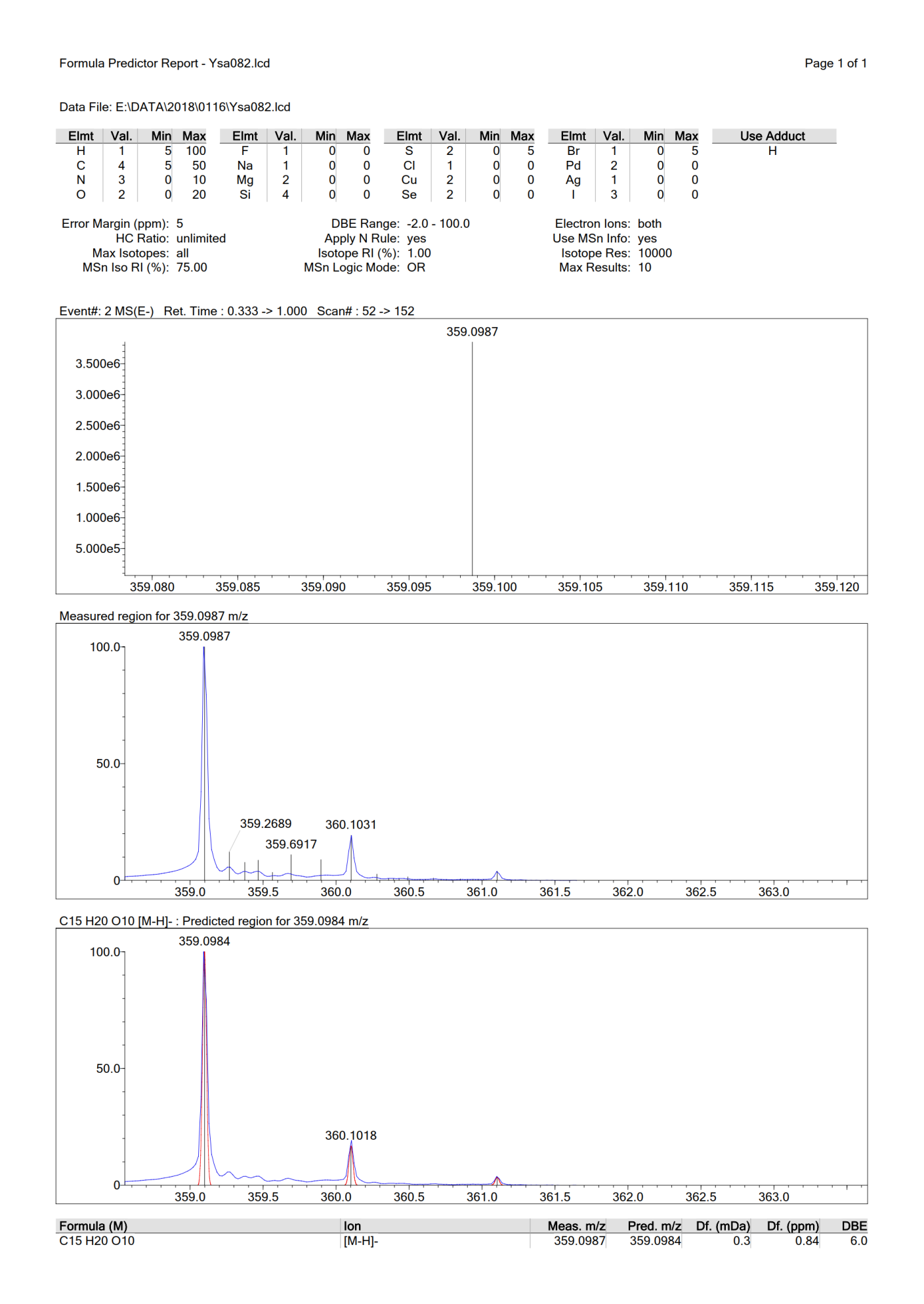


**S16** HRESI spectrum of compound **2**


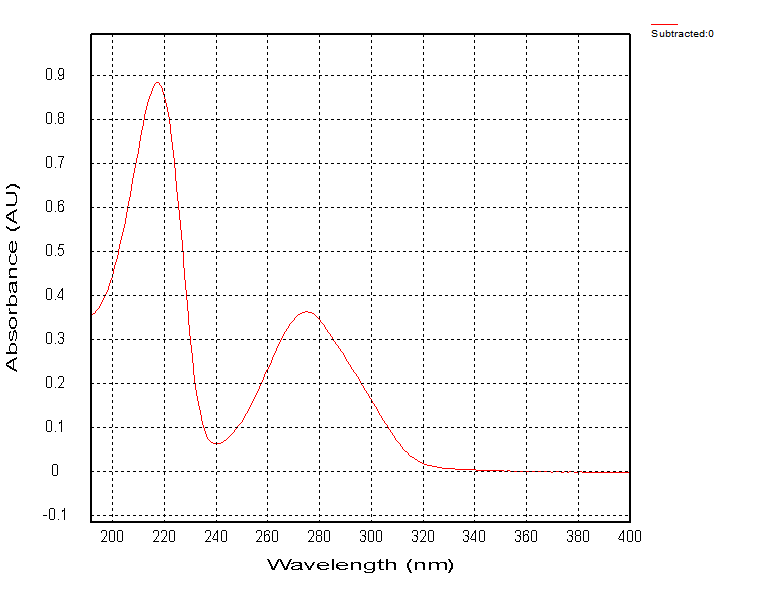


**S17** UV spectrum of compound **2**


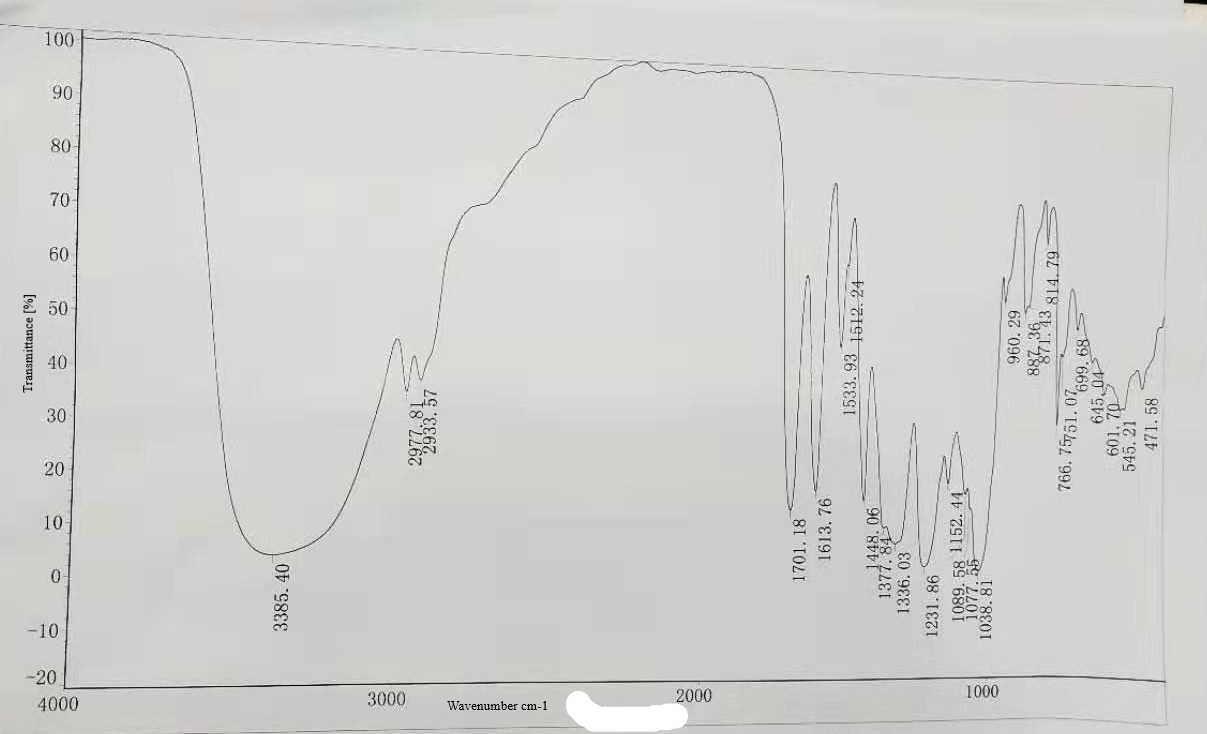


**S18** IR spectrum of compound **2**
